# Apolipoprotein E impairs aged bone fracture healing

**DOI:** 10.1093/geroni/igab046.2499

**Published:** 2021-12-17

**Authors:** Abhinav Balu, Rong Huang, Kristin Molitoris, Gurpreet Baht

**Affiliations:** 1 Duke Molecular Physiology Institute, Durham, North Carolina, United States; 2 Duke University, Durham, North Carolina, United States

## Abstract

Bone fracture healing and osteoblast differentiation are impaired with advanced age. Using a combination of parabiosis and proteomic models, we identified apolipoprotein E (ApoE) to be an aging factor in bone regeneration. Circulating levels of ApoE increased with age in patients and in mice. ApoE impaired bone fracture healing by decreasing bone deposition in the fracture callus which subsequently decreased the mechanical strength of healed tissue. Osteoblasts serve as the sole bone forming cells within the body. In tissue culture models, ApoE treatment decreased osteoblast differentiation and activity which led to decreased matrix formation and mineralization. This inhibition of osteoblast differentiation relied on down-regulation of the Wnt/β-catenin pathway. In mouse models, aged bone repair was rejuvenated when we lowered circulating ApoE levels using a hepatotropic AAV-siRNA model – serving as a proof of concept that ApoE can be targeted to improve bone repair in an older population. While promising, knockdown of circulating ApoE in such a fashion is likely not translatable to patient care. Thus, current work in our laboratory is focused on developing treatment strategies that temporally decrease circulating ApoE levels and consequently improve bone healing after acute injury and/or surgical orthopedic procedure in the geriatric population.

